# Impact of Air Pollution Generated by Brick Kilns on the Pulmonary Health of Workers

**DOI:** 10.5696/2156-9614-11.31.210906

**Published:** 2021-08-17

**Authors:** Ali Raza, Zulfiqar Ali

**Affiliations:** Environmental Health and Wildlife, Department of Zoology, University of the Punjab, Lahore, Pakistan

**Keywords:** respiratory problems, spirometry, lung function, District Kasur, Pakistan

## Abstract

**Background.:**

Brick kiln workers are often not aware of the health effects of their working environment and health-related respiratory problems. There have been few studies on the relationship between brick kiln pollution and its health impact on brick kiln workers.

**Objectives.:**

The present study measured the association of brick kiln contamination with severe respiratory problems and lung function among brick kiln workers in the Kasur district, Pakistan.

**Methods.:**

Air quality variables (PM_2.5,_ PM_10_, sulfur dioxide (SO_2_), nitrogen dioxide (NO_2_) and volatile organic compounds (VOCs)) were monitored during operations in brick kiln modulation and kiln areas. Workers (n=60) were selected for participation if they were between the ages of 18 and 60 and had been working in brick kilns for at least one year and gave consent to participate. Their lung function was measured.

**Results.:**

The average concentrations of measured air quality variables for all working sites were found to exceed the World Health Organization (WHO) and National Ambient Air Quality Standard (NAAQS) guidelines. These high values of brick kiln pollutants were associated with a significant decrease in spirometric values (forced vital capacity (FVC), forced expiratory volume in one second (FEV1), peak expiratory flow (PEF), and average flow between 25% and 75% of the FVC (FEF2575)) among workers and revealed that 78.33% of workers had abnormal lung function with 5% obstructive and 95% restrictive impairments. Occurrences of pulmonary problems like frequent cough (50%), chronic cough (11.67%), frequent phlegm (21.67%), chronic phlegm (11.67%), frequent wheezing (20%), chronic wheezing (15%), shortness of breath grade-I & grade-II (38.33%) and self-reported asthma (3.33%) were also found among the workers.

**Conclusions.:**

Pollution from brick kiln operations was significantly high and associated with respiratory problems as well as a decrease in lung function. There was a clear correlation between pulmonary function in workers with brick kiln contamination.

**Participant Consent.:**

Obtained

**Ethics Approval.:**

This study was approved by the Bioethics Committee of the Department of Zoology, University of the Punjab, Lahore, Pakistan (Ref.1443-UZ).

**Competing Interests.:**

The authors declare no competing financial interests.

## Introduction

In Pakistan, workers at brick kilns are often not aware of the health effects of their working environment and occupational respiratory problems. Owing to lack of education and illiteracy, workers often do not access proper health care facilities due to high costs as well as inaccessibility. Brick kiln workers are primarily exposed to heat and air pollution at the worksite and housing in the vicinity of kilns.^1^

In low- and middle-income countries, brick production is an energy-intensive process, with fossil fuels and wood burning playing an important role in the formation of air pollution.^2^ The use of low-quality coal and other fuels during the brick firing process is the biggest source of harmful emissions from brick kilns.^3^ Brick kiln processes and flue gases are mainly composed of fly ash, sulfur dioxide (SO_2_), carbon dioxide (CO_2_), nitrogen oxide (NO_X_), carbon monoxide (CO), volatile organic compounds (VOCs) and particulate matter, which is often toxic and found to be above from the existing World Health Organization (WHO) and National Ambient Air Quality Standard (NAAQS) guidlines.^4^

Excessive amounts of pollutants and gases are dangerous to humans and can cause respiratory problems. Among the different types of brick kiln workers, modulators, firemen, loaders and unloaders have the highest risk of contamination.^1^,^5^–^9^ Inhalation of these pollutants causes skin and eye irritation and can cause intestinal infections, diarrhea, asthma, bronchitis, cough, pharyngitis, pulmonary fibrosis, emphysema, allergic rhinitis and decline in lung function as well as low birth weight.^10^–^12^

There are an estimated 300 000 brick kilns worldwide, 75% of which are in Pakistan, India, China and Bangladesh.^13^ Pakistan is the third largest producer of bricks,^14^ with about 7000 brick making units in operation, employing about 100 000 permanent workers.^15^ Of the thousands of brick-making units in the country, about 5000 are located in cities and towns in the Punjab province.^16^ The Kasur district has the highest number of brick kilns (352), according to data compiled by the Labor and Human Resource Department, Government of Punjab, Pakistan.^17^

Keeping in view the limited available data, the current study aimed to determine the levels of pollution generated by brick kilns and the effect on kiln worker pulmonary health. The main objectives were to determine the concentration of workplace brick kiln air pollution, to assess the signs and symptoms of respiratory problems among brick kiln workers, and to evaluate pulmonary function variables.

**Abbreviations**
*FEF*_*25*_: 25% flow of FVC
*FEF*_*2575*_: Average flow between 25% and 75% of the FVC
*FEF*_*59*_: 50% flow of FVC
*FEF*_*75*_: 75% flow of FVC
*FEV*_*1*_*%*: Percentage of forced expiratory volume
*FEV*_*1*_: Forced expiratory volume in one second
*FVC*: Forced vital capacity
*PEF*: Peak expiratory flow

## Methods

**Figure 1:**
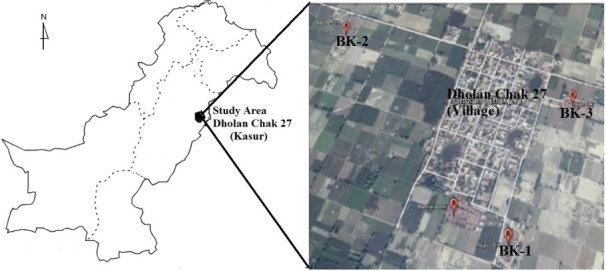
Location of study area within Pakistan indicating brick kiln locations

The current study was conducted from January to April 2018 among brick kilns located at Dholan chak 27 (situated at an altitude of 189 m, 73^o^E longitude and 31^o^N latitude), Pattoki (district Kasur), Pakistan. Three brick kilns in the study area were selected and identified as BK-1, BK-2 and BK-3. There were no other pollution-emitting industrial units present in/around 5 km of study area except for the brick kilns *(Figure 1).*

**Figure 2:**
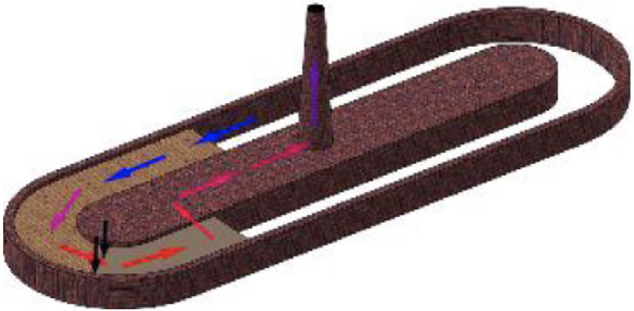
Sketch of a fixed chimney bull trench kiln

The selected brick kilns (n=3) were chosen according to the following criteria: rural location, ≥ 50 employees at each brick kiln, production ≥ 10 million bricks per annum, and coal consumption ≥ 200 000 kg. The brick kilns in the study area were continuous fire kilns in which a fire burns and moves in a closed circuit through bricks stacked in a trench *(Figure 2).*

**Figure 3:**
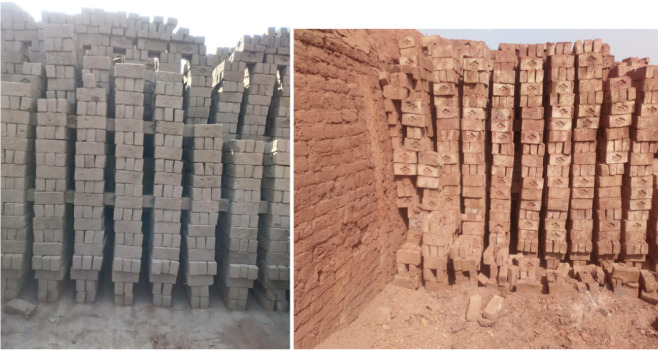
Arrangement of bricks at brick kiln loading site (left) and unloading site (right).

**Figure 4:**
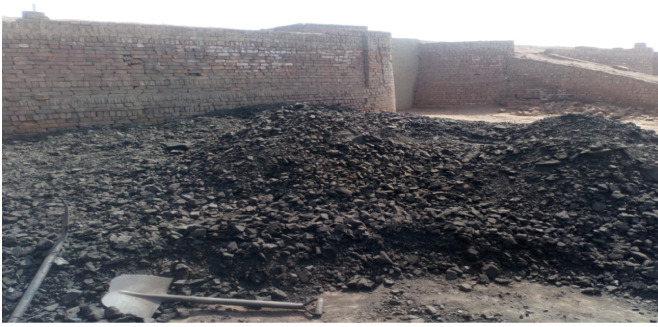
Coal used for firing bricks at brick kilns

The bricks were arranged in the kiln in the order of “column blade” bricks in which bricks were laid in vertical columns along the width of the trench. Rows of brick columns were arranged in the direction of the air flow, one in front of the other *(Figure 3).* The main fuel used in selected brick kilns was low grade coal (anthracite) *(Figure 4).*

### Air quality monitoring

Real-time monitoring of particulate matter less than 2.5 microns in diameter (PM_2.5_), particulate matter less than 10 microns in diameter (PM_10_), nitrogen dioxide (NO_2_), sulfur dioxide (SO_2_), volatile organic compounds (VOCs), temperature and humidity were monitored during 8 hours of operation from 8:00 am to 4:00 pm and data were collected after each hour. A portable dust particle counter (Dylos DC-1700), factory calibrated, was used for measuring PM_2.5_ and PM_10_ particles. The number of particles was later converted to mass concentration (μgm^−3^) using a Dylos conversion sheet (Microsoft Excel) following Equation 1:

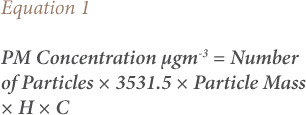



Where H is the relative humidity percentage, C is the correction factor, the mass of a particle in the PM_2.5_ channel is 5.89 × 10^−7^μg, the mass of a particle in the PM_10_ channel is 1.21 x10^−4^ μg and the constant in formula (3531.5) is used for the conversion of particles in ft^3^ to particles in m^3^.^18^

A portable gaseous pollution sampler (Aeroqual-500), with factory calibrated sensors was used to monitor concentrations of NO_2_, SO_2_, VOCs, temperature and humidity. Air quality measurements were performed in regular intervals by placing the equipment in working sites (modulation area and near burning sites of kiln area) of brick kilns. The monitoring point was selected by determining the working situation of each brick kiln site.

### Participant selection and grouping

First, the brick kilns were visited and approval was sought from their owners to collect worker data. The study was approved by the Bioethics Committee of the Department of Zoology, University of the Punjab, Lahore, Pakistan (Ref.1443-UZ). Workers at selected brick kilns were verbally informed about the research and written consent for participation was taken. A greater number of male workers agreed to participate in the study compared to female workers. Study participants were placed into two groups on the basis of their working sites: modulation area and kiln area. Modulation area workers were mainly exposed to dust, but sometimes were also exposed to pollutants from the kiln chimney, whereas workers in the kiln area were exposed to kiln chimney pollutants as well as dust and high temperatures. The main activities of the workers in the modulation area were digging, wetting and mixing clay to form mud, lifting mud, molding bricks and arranging bricks for drying under the sun. Workers in the kiln area carried, loaded and arranged the clay bricks, added coal to the kiln for firing the clay bricks, unloaded and sorted the fired bricks.

### Ethics approval

The study was approved by the Bioethics Committee of the Department of Zoology, University of the Punjab, Lahore, Pakistan (Ref.1443-UZ).

### Questionnaire

A questionnaire was designed for collecting information about worker occupational history and pulmonary health *(Supplemental Material).* Data were collected from 60 male brick kiln workers between 18 and 60 years of age. No females agreed to participate. The questionnaire consisted of five sections collecting information on name, gender, age, weight, height, job categories, working experience, working duration, smoking habit information and pulmonary health problems.

### Spirometery

A portable electronic handheld spirometer (SP 10 Spiroton MDX Instruments USA) with a disposable mouthpiece was used for spirometery. Gender, age, height, weight, smoking and drug use habits of participants was recorded. The spirometry was repeated two to three times with each worker to get accurate data. The forced vital capacity (FVC), forced expiratory volume in one second (FEV_1_), FEV_1_/FVC ratio, peak expiratory flow (PEF), 25% flow of FVC (FEF_25_), 75% flow of FVC (FEF_75_) and average flow between 25% and 75% of the FVC (FEF_2575_) were recorded in liters and percentages and interpreted with the help of respiratory experts.

An international method of measuring lung function was used that helps to detect the presence or absence of abnormalities related to restrictive or obstructive impairments.^19^ The predicted percentages of FVC ≥80% as well as FEV_1_ and the FEV_1_/FVC ratio ≥0.7 were interpreted as normal lung function, while the predicted percentage of FVC <80% as well as FEV_1_ and FEV_1_/FVC ratio <0.7 were considered to indicate abnormal lung function.^20^ In addition, predicted values of FEV_1_ <80% and FEV_1_/FVC <70% indicated obstructive lung function, while the predicted values of FEV_1_ <80% and FEV_1_/FVC >70% indicated restrictive lung function.^21^

### Descriptive statistics

Quantitative variables like age, body mass index, nature of the job, job experience, working duration (hours), smoking habit, intensity of smoking habit and respiratory disease symptoms, spirometric values and air quality variables were presented in frequency tables and interpreted directly using the Statistical Package for the Social Sciences (SPSS) version 20.

## Results

Among workers who consented to participate (n=60), 78.33% of workers had abnormal lung function, with 5% obstructive and 95% restrictive impairments. Smoking was reported by 41.6% of the workers in the present study, while 58.4% were non-smokers. The majority of the workers were aged between 25–30 years with varying years of experience at the brick kilns. Twenty-five percent (25%) of the selected workers worked in the modulation area, while 75% were involved in loading, burning and unloading of the bricks at kiln area. Moreover, 73% of these workers worked eight hours daily, while 27% of workers spent 10 hours on average at their worksite. Around 80% of workers were observed to have a normal body mass index, while obesity was uncommon 3.3% *(Table 1).*

**Table 1:** Percentages of socioeconomic parameters of brick kiln workers

**Age (Years)**	**% of participants**	**Body Mass Index**	**% of participants**	**Years working**	**% of participants**
18–24	18.33	Underweight group (BMI <18.5)	8.33	1–5	3.33
25–30	28.33	Normal weight group (18.5 ≤ BMI < 24)	80	6–10	56.67
31–36	10	Overweight group (BMI ≥ 24)	8.33	11–15	18.33
37–42	13.33	Obese group (BMI ≥30).	3.33	16–20	15
43–48	11.67			More than 20	4
49–54	13.33				
55–60	5				

Abbreviation: BMI, Body Mass Index

Table 2 presents the representative values of different pollutants monitored at the selected brick kilns, while Table 3 presents data on the frequency and percentage of self-reported pulmonary health problems among brick kiln workers. Table 4 describes pulmonary function with reference to smoking habits and Table 5 indicates the pulmonary function of workers in the kiln and modulation areas.

**Table 2:** Air Quality Variables at Brick Kiln Areas

**Brick kiln areas**		**Monitored air quality variables**						

		PM_2.5_ (μgm^−3^)	PM_10_ (μgm^−3^)	SO_2_ (ppm)	NO_2_ (ppm)	VOCs (ppm)	Temp (°C)	RH (%)
**Modulation**	BK 1	19.54	339.02	0.00	0.0565	1250.29	34.85	33.83
	BK 2	56.30	668.36	0.00	0.0476	1715.18	32.33	34.86
	BK 3	69.89	961.69	0.00	0.0731	943.76	36.16	23.33
	Mean ± SD	48.58 ± 26.05	656.36 ± 26.05	0.00 ± 0.00	0.0591 ± 0.013	1303.08 ± 388.41	34.45 ± 1.95	30.67 ± 6.38

**Kiln**	BK 1	117.27	1468.40	0.0436	0.068	1437.65	37.64	30.98
	BK 2	114.80	1626.48	0.0756	0.064	1118.71	35.52	33.30
	BK 3	68.94	887.77	0.0763	0.077	761.76	35.52	24.44
	Mean ± SD	100.34 ± 27.22	1327.55 ± 388.98	0.0652 ± 0.018	0.070 ± 0.007	1106.04 ± 338.12	36.23 ± 1.22	29.57 ± 4.59

Abbreviations: BK, brick kiln; VOCs, volatile organic compounds; RH, relative humidity; SD, standard deviation

**Table 3:** Frequency and Percentage of Self-reported Pulmonary Health Problems among Brick Kiln Workers

**Self-reported pulmonary health problems**	**Frequency**	**% of participants**
Frequent cough	30	50
Chronic cough	7	11.66
Frequent phlegm	13	21.66
Chronic phlegm	7	11.66
Frequent wheezing	12	20
Chronic wheezing	9	15
Shortness of breath Grade I and Grade II	23	38.33
Self-reported asthma	2	3.33
Physician-diagnosed asthma	0	0

**Table 4:** Descriptive Statistics of Spirometric Pulmonary Function with Reference to Smoking Habit

**Variables**	**Smoking habit**			

	**Non-smokers (n=35)**		**Smokers (n=25)**	

	**Mean**	**±SD**	**Mean**	**±SD**
FVC (liter)	2.53	0.736	2.27	0.670
FEV_1_ (liter)	2.16	0.616	1.98	0.534
PEF (1/s)	4.12	2.055	3.75	1.482
FEV_1_ %	86.83	13.870	89.24	14.420
FEF_2575_ (l/s)	2.66	1.029	2.61	1.087
FEF_25_ (l/s)	3.47	1.587	3.32	1.367
PEF_75_ (l/s)	1.80	0.766	1.84	0.860

Abbreviations: FVC, forced vital capacity; FEV_1_ forced expiratory volume in one second; PEF, peak expiratory flow; FEV_1_%, percentage of forced expiratory volume; FEF_2575_, average flow between 25% and 75% of the FVC; FEF_25_, 25% flow of FVC; FEF_50_, 50% flow of FVC; 1/s, liter per second; SD, standard deviation

**Table 5:** Descriptive Statistics of Pulmonary Function of Workers in Kiln and Modulation Areas

**Variables**	**Kiln area (n=45)**		**Modulation area (n=15)**	

	**Mean**	**±SD**	**Mean**	**±SD**
FVC (liter)	2.42	0.74	2.45	0.67
FEV_1_ (liter)	2.09	0.63	2.09	0.44
PEF (l/s)	4.16	2.03	3.37	0.86
FEV_1_%	88.04	14.34	87.20	13.54
FEF_2575_ (l/s)	2.68	1.13	2.53	0.76
FEF_25_ (l/s)	3.52	1.64	3.07	0.88
PEF_75_ (l/s)	1.85	0.84	1.72	0.68

Abbreviations: FVC, forced vital capacity; FEV_1_, forced expiratory volume in one second; PEF, peak expiratory flow; FEV_1_%, percentage of forced expiratory volume; FEF_2575_, average flow between 25% and 75% of the FVC; FEF_25_, 25% flow of FVC; FEF_50_, 50% flow of FVC; 1/s, liter per second; SD, standard deviation

## Discussion

The aim of the current study was to estimate the levels of PM_2.5_, PM_10_, SO_2_, NO_2_ and VOCs generated by brick kilns in selected sites of Tehsil Pattoki and their effect on the pulmonary health of brick kiln workers. The results of the current research indicated levels of selected pollutants measured from kiln and modulation areas of brick kilns to be many times higher than the prescribed limits*.* According to National Ambient Air Quality Standard (NAAQS) guidelines, the average concentrations for PM_2.5_, PM_10_, SO_2_ and NO_2_ should not exceed 35 μgm^−3^, 150 μgm^−3^, 0.5 ppm and 0.053 ppm, respectively.^22^ Likewise, a maximum annual daily mean exposure of 20 μg/m^3^ and a maximum 24-hour mean exposure of 50 μgm^−3^ for PM_10_ were suggested by the World Health Organization (WHO) in order to reduce harmful health outcomes linked with particulate matter air pollution.^23^ In comparison with these levels, kiln emissions were many times higher.

Similar results were found in a previous study where average concentrations of PM_2.5_ in the modulation, burning and unloading sections were 301 μgm^−3^, 307 μgm^−3^ and 628 μgm^−3^, respectively, whereas the average concentrations of PM_10_ in modulation, burning and unloading sections were 888 μgm^−3^, 1830 μgm^−3^ and 861 μgm^−3^, respectively.^9^ Likewise, another study observed the average level of PM_10_ in an area of kiln operations to be 415 μgm^−3^, which is less than the current recorded value (1327.55 μgm^−3^).^24^ In another study, mean concentrations of 480 μgm^−3^ and 172 μgm^−3^ were determined for PM_10_ and PM_2.5_, respectively.^25^ Very high concentrations of respirable dust (19 510 μgm^−3^ in the kiln section and 10 080 μgm^−3^ in the modulation section) were observed as well.^26^

In addition, an average PM_10_ concentration of 29 μgm^−3^ was recorded prior to brick kiln operations and increased up to 50 μgm^−3^ during the period of operations, which were very low values for PM_10_ compared to current studies.^2^ Similarly, in another study, the concentration of PM_10_ for the pre-operation period was reported to be 29 μgm^−3^, but increased up to 50 μgm^−3^ for the period of brick industry operations.^3^

Other than particulate matter, levels of gaseous pollutants have also been reported to vary across different studies. In a recent study, the respective SO_2_ levels at three different sites of brickfield of district Budgam, Jammu and Kashmir, were reported to be 0.047 ppm, 0.044 ppm and 0.037 ppm.^3^ Another study revealed that SO_2_ values were in the range 0.003-0.01 ppm during the pre-production period and 0.010–0.032 ppm during the period of brick production at kiln areas of brick kilns cluster in Tripura, India and these values were relatively lower^27^ compared to the kiln areas of present study. Similar to the results of the current study, the concentration of NO_2_ was 0.058, 0.052 and 0.041 ppm at kiln areas of three different brick kilns located at district Budgam of Jammu and Kashmir.^24^ In contrast to the present study, much lower values of NO_2_ ranging from 0.005–0.009 ppm during the pre-production period and 0.008–0.018 ppm were reported during brick production at kiln areas of brick kilns cluster in Tripura, India.^27^

Apart from particulate and gaseous pollutants, humidity and temperature were also important factors affecting the health and comfort level of workers. The temperature of the kiln area was higher than the temperature in the modulation area whereas the relative humidity was higher in the modulation area than the kiln area*.* Similar results were also reported during the study of brick kilns of west Bengal, India.^28^

In light of the health impact of exposure to elevated levels of pollutants, it was hypothesized that brick kiln workers would be at risk of respiratory disorders as reported by previous studies. Similar to the current study, increased incidence of pulmonary problems, ie. phlegm, cough, asthma, wheezing and breathlessness among brick kiln employees was reported in a cross-sectional study conducted at India.^26^ Another study reported a significant occurrence of 31.8% chronic cough, 24% chest tightness and 26.2% chronic phlegm in brick kiln employees.^29^ The most commonly reported pulmonary problems among brick kiln workers were chronic cough (34.70%), dyspnea/shortness of breath or breathlessness (21.4 %), chest wheeze (20.2%), chronic bronchitis (19.7%), and asthma (15.63%).^30^ A survey reported 32%, 24%, 15%, 28%, and 11% occurrence for chronic cough, chronic phlegm, chest wheeze, dyspnea and asthma, respectively,^26^ while another study reported the major respiratory symptom among brick kiln workers to be 19% phlegm, 17.5% cough, 14% wheeze, 10.5% breathlessness, 9.5% self-reported asthma and 5.5% physician diagnosed asthma.^31^ The existence of pulmonary problems was associated with obstructive lung function and high occurrences of pulmonary problems (31.8% chronic cough, 26.2% chronic phlegm and 24% chest tightness) were observed in brick kiln workers.^32^ The present study found that mean spirometric values were significantly decreased and decreased levels of the measured pulmonary variables have been reported in previous studies as well.^28^,^32^–^33^

The mean spirometric values of FVC, FEV_1_, PEF, FEF_2575_ and FEF_25_ were decreased among smokers as compared to non-smoker workers of the present study*.* Similarly, the results of related studies showed significant decreases in PEFR, FEV_1_, FVC, FEF (25–75%) in workers who were also smokers.^34^–^35^ In parallel to current findings, lung function values were significantly reduced among smokers compared to non-smokers.^28^,^36^

In the present study, there were considerable differences among the spirometric values of FVC, FEV_1_, PEF, FEV_1_% and FEF_2575_ of workers who reported working shifts of ≥8 hours and ≥10 hours, indicating that worker lung function was decreased with increasing working hours. Similar findings were reported in other studies where increasing working hours were associated with reduced lung capacity of brick kiln workers.^9^,^37^ Similar to the current study, several studies indicated that the mean values of FVC, FEV_1_ and FEV_1_/FVC ratio were significantly decreased in brick kiln workers.^9^,^32^,^33^,^37^

In the present study, brick kiln workers were found to be at risk of obstructive as well as restrictive impairments, which has been confirmed by previous studies.^31^,^34^ The current results were also confirmed by a study on female brick kiln molders that suffered from chronic obstructive pulmonary disease.^8^ Another study found that the prevalence of COPD among brick kiln workers was 18.9%.^38^ Various other studies supported the current results of significantly reduced FVC and FEV_1_ among brick kiln workers, indicating restrictive lung function.^9^,^32^,^39^

Workers from various brick kiln workplaces were not aware of the health risks in the current study, as confirmed by previous research in Okara, Pakistan.^40^ In the present study, workers did not wear masks or any other personal protective equipment (PPE). Previous studies have reported that brick kiln workers often do not use safety precautions.^41^–^42^

### Limitations

In the present study, air quality monitoring was limited to areas where work was in progress and wind speed data were not measured. To overcome this limitation, future research should select different sites to monitor air quality as well as wind speed around the brick kilns. Also, some workers were exposed to smoke from wood burning for cooking at their residences. All study participants were male.

## Conclusions

A high frequency of respiratory symptoms and diseases were observed in brick kiln workers. Age, type of work, working hours and smoking were strongly associated with respiratory symptoms and disease development. There was a significant association between exposure to pollutants in the workplace and impaired lung function in brick kiln workers. In the present study, 21.66% of workers were healthy with normal lung function and 78.33% had abnormal lung function, with 5% obstructive and 95% restrictive impairments. Moreover, brick kiln workers were not observed to use any PPE.

In light of the pollution generated by brick kilns, it is important to focus on reducing kiln emissions. Poorly designed kilns along with combustion of coal and wood in results in high and potentially hazardous levels of particulate matter and VOCs which threaten the health of brick kiln workers.
