# Supplementary material for: Impact of Air Pollution Generated by Brick Kilns on the Pulmonary Health of Workers
Source: J Health Pollut. 2021 Aug 17;11(31):210906. doi: 10.5696/2156-9614-11.31.210906 (PMC8383793; doi:10.5696/2156-9614-11.31.210906)
Supplement: Supplementary file 1 [file Ali_Supplemental_Material.docx]

**Supplemental Material**

**Participant information sheet**

**Background**

Air pollution is the biggest threat to human health. A large amount of air pollution is being emitted from brick kilns and causing environmental and health problems in the surrounding area. There have been numerous studies carried out concerning environmental pollution of brick making industries, but few studies have described the effects of brick kiln pollution on the pulmonary health of workers. Unfortunately, this field has often been ignored in Pakistan.

**Study objective**

The main objective of this study is to examine the relationship between pulmonary health problems of workers and their exposures to pollution from different brick kiln processes.

**Procedure**

You will be asked about your workplace air quality by questionnaire after the study is fully explained. Moreover, one of the following variables will be monitored:

- Weight and height measurements
- Spirometry

**Possible risks or discomforts**

There will not be any possible known risk to the patient.

**Possible benefits**

You will be informed about workplace air pollution levels and the results of testing related to your health. In addition, this study will be informative for workers of brick kilns and the people living in vicinity of brick kilns.

**Financial considerations**

All the financial costs of this research will be paid by the researcher. You will not incur any kind of cost or expense**.**

**Medical treatment for adverse experiences**

You will be provided medical treatment by the researcher in case of any adverse event or discomfort.

**Confidentiality**

All the information related to you will be treated as strictly confidential and will only be available to investigators involved in this study.

**Termination of participation**

Participation in this study depends on your consent. There will be no penalty if you choose not to participate further at any stage of the study.

**Further information**

Any further questions you have about the study will be answered by the researcher.

**Consent Form**

- I have read and understood this consent form or this consent has been fully explained to me by the researcher, and I voluntarily participate in this research study.
- I understand that I will receive a copy of this form. I voluntarily choose to participate.
- I further understand that nothing in this consent form is intended to replace any applicable federal, state or local laws.

Participant’s name in full: ------------------------ Name of investigator: --------------

Signature or thumb impression of Participant

------------------------- Date: ------------------

Date: -------------------------

**Questionnaire**

**Survey ID: ______________ Date: _______________**

**SECTION I**

**General information**

**1.** Name of the interviewee: ___________ **2.** Gender: Male/Female

**3.** Age: Years ____________ **4.** Telephone#: ____________

**4.** Address________________________________________________________________

**SECTION II**

**General health information**

**1.** Weight ________kg **2.** Height _____________cm

**SECTION III**

**Work-related information**

**1.** Job categories:

**a)** Modulation worker **b)** Loading worker **c)** Burning worker **d)** Unloading worker

**2.** Name of brick kiln where you work________________________________

**3.** Work experience in years***________________***

**4.** How long do you work each day on average? ___________hours

**5.** Do you wear a dustproof mask when working? **a)** Yes **b)** No

**6.** Do you wear tight-fitting eyeglasses when working? **a)** Yes **b)** No

**7.** Do you wear a coverall when working? **a)** Yes **b)** No

**8.** Do you wear gloves when working? **a)** Yes **b)** No

**SECTION IV**

**Smoking habit**

Are you a smoker or non-smoker?

**a)** Non-smoker

**b)** Smoker

**SECTION V**

**Health problems**

Do you often suffer from or have you ever had any of the following symptoms?

| **Health problems** | **Yes** | **No** |
| --- | --- | --- |
| Frequent cough |  |  |
| Chronic cough |  |  |
| Frequent phlegm |  |  |
| Chronic phlegm |  |  |
| Frequent wheezing |  |  |
| Chronic wheezing |  |  |
| Shortness of breath Grade I and Grade II |  |  |
| Self-reported asthma |  |  |
| Physician-diagnosed asthma |  |  |
